# Impact of the Dietary Fat Concentration and Source on the Fecal Microbiota of Healthy Adult Cats

**DOI:** 10.3390/metabo15040215

**Published:** 2025-03-22

**Authors:** Nadine Paßlack, Kathrin Büttner, Wilfried Vahjen, Jürgen Zentek

**Affiliations:** 1 Small Animal Clinic, Faculty of Veterinary Medicine, Justus-Liebig-University Giessen, 35392 Giessen, Germany; 2 Unit for Biomathematics and Data Processing, Justus-Liebig-University Giessen, 35392 Giessen, Germany; kathrin.buettner@vetmed.uni-giessen.de; 3 Institute of Animal Nutrition, Department of Veterinary Medicine, Freie Universität Berlin, 14195 Berlin, Germany; wilfried.vahjen@fu-berlin.de (W.V.); juergen.zentek@fu-berlin.de (J.Z.)

**Keywords:** microbiome, short-chain fatty acids, lactate, ammonium, feces, feline, oil, lard

## Abstract

Background/Objectives: The dietary fat supply might interact with the intestinal microbiota via different mechanisms. Research on this topic, however, remains scarce in cats. For this reason, the present study was conducted to evaluate the impact of the fat concentration and fatty acid profile in the diet on the fecal microbiota of healthy cats. Methods: A low-fat basal diet was fed to ten healthy adult cats. The diet was offered without or with the daily addition of 0.5 g or 1 g of sunflower oil, fish oil or lard per kg body weight of the cats, using a randomized cross-over design. Each feeding period lasted for 21 days, and the fecal samples were collected on the last days of each period. The fecal microbiota was analyzed by 16S rDNA sequencing. Additionally, microbial metabolites (short-chain fatty acids, lactate, ammonium, biogenic amines) were measured in the fecal samples. Results: The dietary treatment had no impact on the alpha-diversity of the fecal microbiota or on the relative abundance of bacterial phyla in the samples. Only a few changes were observed in the relative abundance of bacterial genera and the concentrations of microbial metabolites in the feces, probably being of minor physiological relevance. Conclusions: The balanced intestinal microbiota of cats seems to be relatively resistant to moderate variations in the dietary fat supply over a short feeding period. Longer-term treatments and higher dietary fat levels should be evaluated in future studies to further clarify the relevance of fat intake for the feline gut microbiome.

## 1. Introduction

The diet is a significant modulator of the intestinal microbiota [1]. In cats, for instance, it has been demonstrated that the dietary protein level and source [2,3,4,5,6], dietary fibers (reviewed by [7]), or the starch content in the diet [8,9,10,11] can influence the composition and metabolic activity of the gut microbiome. However, while data from other species, such as humans (reviewed in [12,13,14,15,16,17]) and dogs [18,19,20], indicate that the dietary fat supply can also be a relevant factor to affect the intestinal microbiota, little is known about this relationship in felines.

The potential mechanisms of how the fat content of a diet might interact with the gut microbial community include antibacterial or bacteria-promoting effects [17]. In addition, it is discussed that dietary fatty acids modulate immunological pathways first, which can then also impact the intestinal microbiome [13,15]. The mechanisms involved are not yet fully understood, but it is assumed that not only the fat concentration in a diet, but also its fatty acid profile, i.e., the saturation, chain length and double bond position of fatty acids, are the relevant factors that mediate the fat’s impact on gut microbes [17].

In dogs, a recent meta-analysis revealed the highest alpha-diversity of the fecal microbiota when moderate-fat diets (15 to ≤20% crude fat in dry matter) were fed [20]. Moreover, the richness of the microbiota was the highest when low-fat diets (5 to ≤15% crude fat in dry matter) were offered to the dogs. The study could further demonstrate differences in the bacterial phyla and genera in the feces of the animals depending on the dietary fat concentration [20].

Whether such effects of the dietary fat content on the gut microbiome might also be observed in cats has been scarcely studied so far. Only one investigation is available in this context, evaluating not only the varying fat levels, but also different carbohydrate sources [10]. The results revealed that both dietary factors modulated the fecal microbiota of cats, even after a relatively short feeding period of 28 days [10].

From a practical point of view, dietary fat intakes in cats can vary widely. The data from cats, and even more so the results from other species, indicate that the fat supply might interact with the intestinal microbiome; however, more targeted studies are required to conclude on the relevance of dietary fat for the feline microbial community, its metabolism, and finally the gut health of the host. Therefore, the aim of this study was to evaluate the impact of varying fat concentrations in a diet (9–15% on a dry matter basis) as well as of different dietary fatty acid ratios (high in either n-6, n-3 or saturated fatty acids) on the fecal microbiota of cats in more detail.

## 2. Materials and Methods

The present investigation received approval by the ethics committee of the relevant local authority (“Regierungspräsidium Giessen”, Giessen, Germany; ethical approval code G41/2022).

### 2.1. Study Design

For this randomized cross-over study, 7 neutered female and 3 neutered male healthy European Shorthair cats were used. At the beginning of the investigation, the animals were aged 46.6 ± 14.1 months and showed a body weight of 4.99 ± 0.91 kg. The cats were housed in the research facility of the Small Animal Clinic of the Justus-Liebig-University Giessen, Germany, either as one group during the adaptation periods or individually in metabolic cages (length: 1.7 m, width: 0.9 m, height: 1.25 m; additional second floor of 0.6 m^2^) for the feces collection.

The cats were fed a commercial complete low-fat diet (Vet-Concept, Cat Intestinal Low Fat, Föhren, Germany) as a basal treatment throughout the study period. The analyzed crude fat content of the diet was 9% in dry matter (Table 1). In total, the study consisted of 7 feeding periods of 3 weeks each, where the basal low-fat diet was fed without or with the addition of sunflower oil (Aro, MCC Trading International GmbH, Düsseldorf, Germany), fish oil (Grizzly Lachsöl, Grizzly Pet Products, Woodinville, WA, USA) or lard (private butcher in Hesse, Germany). The oils or lard were added daily to the diet at 0.5 g or 1 g per kg body weight of the cats, as also recently described [21]. By this, dietary crude fat concentrations of approximately 12% and 15% in dry matter were achieved. The fatty acid patterns of the diet, oils, and lard are presented in Table 2.

The cats were randomly assigned to the dietary treatments, using a modified Latin square design. As the amount of lard from one batch was limited, two cats could not receive the treatment “1 g lard/kg body weight/day”. To avoid any impact on the evaluation of dose effects, the same fat supplement was not used in two consecutive feeding periods [21].

Fecal samples were collected on the last five days of each feeding period and stored at −80 °C until further analyses. The samples were intended for nutrient analyses, as published before [21]. For the analysis of the microbiota and microbial metabolites, samples obtained on the last day of each feeding period (day 21) were used. If a cat did not defecate on that day, the sample from the preceding day with defecation was considered (mostly day 20 and in one case day 19).

The daily amounts of food were calculated to maintain the body weight of the cats [22]. The feed intake of the animals was assessed daily, and the body weight was assessed weekly.

### 2.2. Measurement of the pH and Microbial Metabolites in the Fecal Samples

The pH in the feces of the cats was measured with a pH meter (Seven Multi pH meter, Mettler-Toledo GmbH, Schwerzenbach, Switzerland). The fecal concentrations of short-chain fatty acids were determined with a gas chromatograph (Model 19095 N-123, Agilent Technologies, Santa Clara, CA, USA) and the concentrations of D- and L-lactate using high-performance liquid chromatography (HPLC Agilent 1100, Agilent Technologies, Santa Clara, CA, USA). The ammonium concentrations in the fecal samples were measured colorimetrically with the Berthelot reaction, while the biogenic amines were analyzed using the Biochrom 20 Plus (Laborservice Onken GmbH, Gründau, Germany). More details on the methods are provided in [23,24].

### 2.3. 16S rDNA Sequencing

The relative abundance of the fecal microbiota was assessed using 16S rDNA sequencing, targeting the V3–V4 region of the 16S rDNA gene. The analysis was carried out by the QIIME2 pipeline [25] and the SILVA SSU database [26]. All methods have been described before in detail [27].

Sequencing data on the relative abundance of bacterial genera derived from a number of positive samples that was sufficient for the statistical data analysis are presented in the following tables. Otherwise, they are provided in the Supplementary Material (Table S1).

The raw data are accessible via the BioProject database (http://www.ncbi.nlm.nih.gov/bioproject/1219520, accessed on 19 February 2025).

### 2.4. Statistical Data Analysis

For the descriptive data analysis, SPSS 28 was used (IBM Corp. Released 2021. IBM SPSS Statistics for Windows, Armonk, NY, USA). The group comparisons were carried out with SAS 9.4 (SAS^®^ Institute Inc., 2013. Base SAS^®^ 9.4 Procedures Guide: Statistical Procedures, 2nd edition ed. Statistical Analysis System Institute Inc., Cary, NC, USA). In the first step, a repeated measures ANOVA was used to separately analyze the effects of the 3 dietary treatments (sunflower oil, fish oil, lard), resulting in 3 independent statistical models. For each treatment, 3 doses were considered (no supplementation (w/o), 0.5 g and 1 g of the oil/lard per kg body weight of the cats). In the case of a significant treatment effect, pairwise comparisons were carried out to specify the dose effects (3 comparisons: w/o versus 0.5 g of the oil/lard; w/o versus 1 g of the oil/lard; 0.5 g of the oil/lard versus 1 g of the oil/lard). The pairwise comparisons were adjusted with the Bonferroni correction. Significant dose effects between the groups are marked in the tables with different superscript letters. If no superscript letters appear, the pairwise comparisons did not reveal a statistically significant dose effect.

Additional correlation analyses were carried out for bacterial phyla and genera, but without considering those listed in Table S1 due to the small number of positive fecal samples. The correlation analysis was performed with SPSS 29 (IBM Corp. Released 2021. IBM SPSS Statistics for Windows, Armonk, NY, USA), calculating Spearman’s rho correlation coefficient and the respective *p*-value.

All data are presented in the tables as means and pooled standard error of the means. A *p*-value < 0.05 was considered to be statistically significant and a *p*-value < 0.1 as a trend.

## 3. Results

### 3.1. Health Status, Feed Intake and Body Weight of the Cats

With the exception of one cat, the animals remained healthy during the entire study period. One cat showed respiratory symptoms of a herpes virus infection and was symptomatically treated with infusions, analgesics, inhalation and orexigenic drugs for a few days. The cat was removed from the feeding period “w/o” due to the described medical treatment and also from the consecutive feeding period “0.5 g lard/kg body weight/day” to avoid any impact on the study results. After the recovery, the cat completed the remaining feeding periods without any health problems.

The feed intake and body weight of the animals were not affected by the dietary treatments (Table 3), as recently described in [21].

### 3.2. Fecal pH and Microbial Metabolites

The fecal pH did not differ among the feeding groups. In addition, only small effects of the dietary treatments could be observed for the fecal concentrations of microbial metabolites (Table 4 and Table 5).

The fish oil supplementation affected the i-butyric acid concentrations (*p* = 0.0431) and tended to impact the propionic acid concentrations (*p* = 0.0603) in the feces of the cats. However, the pairwise comparisons could not further specify these effects (*p* > 0.05).

The dietary lard supplementation also modulated the total concentrations and relative amounts of i-butyric acid in the feces (*p* = 0.0332 and *p* = 0.0437, respectively). The group comparisons demonstrated that the i-butyric acid concentrations were lower when 0.5 g lard/kg body weight/day was added to the diet instead of 1 g lard/kg body weight/day (*p* < 0.05). In addition, the relative amounts of i-butyric acid tended to be lower when 0.5 g lard/kg body weight/day were supplemented compared to the control treatment (*p* = 0.0703) and the treatment of 1 g lard/kg body weight/day (*p* = 0.0826).

The inclusion of lard in the diet further tended to influence the concentrations of ammonium (*p* = 0.0837), propionic acid (*p* = 0.0884), n-valeric acid (*p* = 0.0946) and tyramine (*p* = 0.0659) as well as the relative amounts of propionic acid (*p* = 0.0952) and i-valeric acid (*p* = 0.0951) in the feces of the cats.

### 3.3. Alpha Diversity of the Fecal Microbiota

The calculation of the alpha diversity of the fecal microbiota of the cats revealed no differences in the Richness, Shannon Index, or Evenness among the dietary treatment groups (Table 6).

### 3.4. Bacterial Phyla in the Fecal Samples

The dominant bacterial phyla detected in the feces of the cats are presented in Table 7. No differences could be observed based on the supplementation of sunflower oil, fish oil, or lard to the basal diet. The correlation analysis also revealed no dependencies of the bacterial phyla on the dietary fatty acid pattern (Table S2).

### 3.5. Bacterial Genera in the Fecal Samples

Table 8 shows the dominant bacterial genera measured in the fecal samples of the cats. Only a few significant differences could be detected related to the dietary treatment.

An unknown genus of the Family Family XIII was influenced by the dietary supplementation of fish oil (*p* = 0.0169); however, pairwise comparisons did not provide further clarification (*p* > 0.05). The inclusion of lard in the diet modified the relative abundance of *Solobacterium* in the feces of the cats (*p* = 0.0470), while the pairwise comparisons could again not further clarify the group differences (*p* > 0.05). The dietary lard supplementation also influenced an unknown genus of the family *Bifidobacteriaceae* (*p* = 0.0045), with a trend (*p* < 0.1) of a group difference between the control treatment and the lard supplementation at 1 g/kg body weight/day.

A trend for the treatment effect was additionally observed for the relative abundance of *Catenibacterium* (*p* = 0.0734 for the sunflower treatment), *Ruminiclostridium* 9 (*p* = 0.0558 for the sunflower treatment), an unknown genus of the family *Erysipelotrichaceae* (*p* = 0.0844 for the sunflower treatment), *Peptoclostridum* (*p* = 0.0887 for the lard treatment) and *Slackia* (*p* = 0.0994 for the lard treatment).

No significant correlations between the relative abundance of bacterial genera and the dietary fat supply were observed (Table S3).

## 4. Discussion

The present investigation aimed to evaluate the effects of different fat sources and levels in a diet on the composition and metabolic activity of the intestinal microbiota of cats. For this, moderate to higher fat concentrations were achieved by the supplementation of a basal diet with 0.5 g and 1 g oil or lard per kg of the body weight of the animals. While the basal diet was relatively low in fat (9% in dry matter), the addition of the oils and lard resulted in higher dietary fat levels of approximately 12% and 15% on a dry matter basis. In addition to the fat concentration, the dietary fatty acid pattern was also adjusted by the use of sunflower oil (to increase the amount of n-6 fatty acids), fish oil (to provide additional n-3 fatty acids) and lard (to enhance the amount of saturated and monounsaturated fatty acids in the daily meal of the animals).

In general, the intestinal effects of the dietary fat supply might be dependent on the overall fat intake and fat digestibility, since these factors influence the available amount of fatty acids in the gut and their interplay with the microbiome [12]. As previously published, the apparent crude fat digestibility observed in the present study was high (means between 94.0 and 95.6%) and did not differ among the groups [21]. However, due to the variations in the fat intake, the fecal crude fat concentrations increased with the supplementation of sunflower oil, fish oil, and lard [21]. Thus, the different dietary fat doses were accompanied with variations in the fat concentrations in the digesta, which could have potentially influenced the gut microbiome.

The underlying mechanisms of how the dietary fat level or fatty acid profile might affect the intestinal microbiota are not well understood so far [17]. Both antimicrobial and bacteria-promoting effects have been assumed based on the research in human medicine [12,13,15,17]. Those effects are not only related to the total fat intake, but also to the fatty acid composition in the diet. In particular, it has been described that saturated fatty acids decrease the richness and diversity of the intestinal microbiota [28,29,30], while n-3 fatty acids can be able to increase microbial diversity [31] as well as beneficial bacteria, such as Bifidobacteria [32,33,34] or lactic acid bacteria [35,36]. It is important to mention, however, that those studies were not conducted on cats. Since the microbiome differs depending on the animal species [37], dietary interventions might result in a varying response of the host microbiota and, thus, limit the transfer of results from one species to another.

In the present investigation, the dietary fat level and fatty acid pattern only slightly affected the fecal microbiota of cats. The microbiota’s alpha diversity and the relative abundance of dominant bacterial phyla were not changed among the treatment groups. The assessment of the relative abundance of dominant bacterial genera revealed an impact of the dietary lard supplementation on *Solobacterium* and an unknown genus of the family *Bifidobacteriaceae*, while the addition of fish oil to the cats’ diets influenced an unknown genus of the Family Family XIII. As the relative abundance of the three genera, however, was generally low, the relevance of these findings seems to be small.

A few significant differences and trends in the dietary impact on the microbial metabolites in the feces of the cats could be observed. Most of these changes were related to the lard supplementation, i.e., to the provision of saturated fatty acids, while some differences were also detected, when the n-3 fatty acid-rich fish oil was added to the diet. Interestingly, the relative amounts of i-butyric acid in the feces tended to be lower, when 0.5 g lard/kg body weight/day was added to the diet compared to the control treatment without lard. Data from human medicine have also described a reduction in fecal butyrate when diets with saturated fatty acids were ingested [29], but also a general increase in short-chain fatty acids in the feces [38]. With regard to the present data, it should be considered that most pairwise comparisons could not further specify the diet effects observed. Thus, it can only be noted at this point that the oil and especially the lard supplementation has modified the metabolic activity of the gut microbiome to a certain degree. Those effects, however, were not associated with marked changes in the composition of the fecal microbiota of the animals.

Potential mechanisms related to the modulation of the intestinal microbiome by n-3 fatty acids are mainly discussed as resulting from the impact on microbial metabolites or on the immune function [13]. Besides the fact that the present study could only reveal small effects of fish oil supplementation on bacterial metabolites and on the composition of the microbiota in the feces of cats, it should also be noted that no immunological data were evaluated. In general, several studies have demonstrated the immunomodulatory properties of n-3 fatty acids in different species [39,40,41,42,43], including cats [44,45]. Nevertheless, as we did not analyze the immunological impact of the fish oil supplementation in this study, we cannot comment on the potential interaction between the immune system and the gut microbiome of the cats.

The effects of varying dietary fat levels on the intestinal microbiota are sometimes assumed to be a result of concomitant variations in the carbohydrate content of the diet, which impacts the amount of microbially fermentable substrate in the gut [12]. In the present study, however, the oils and lard were supplemented to a basal diet fed to all cats, and the feed intake did not differ among the groups [21]. Thus, with the exception of fat, comparable nutrient intakes can be assumed, implicating that the dietary effects on the intestinal microbiota can be clearly attributed to the variations in the fat concentrations and fatty acid patterns in the daily meals.

Finally, interactions between the gut microbiome and the lipid metabolism of the host are especially investigated in human medicine [14]. For instance, it is discussed that the microbiota can impact the secretion of triglycerides and chylomicrons from enterocytes, which also impacts the circulating concentrations of lipids [14]. In addition, recently published data on lactic acid bacteria isolated from cats indicate lipid-lowering properties of the gut microbiota by cholesterol degradation and antimicrobial effects [46]. As another part of the present feeding trial, we have also evaluated the impact of the dietary sunflower oil, fish oil and lard supplementation on the lipid metabolism of cats [21]. We could demonstrate that the addition of fish oil to the basal diet lowered the concentrations of triglycerides in the plasma, while the total and low-density lipoprotein cholesterol levels were increased. The sunflower oil and lard supplementation revealed no effect on the lipid metabolism of the cats [21]. In light of a potential interaction between the gut microbiota and the lipid metabolism, one might therefore expect that especially the fish oil treatment modified the bacterial community in the feces of the cats. However, the observed effects were only small, questioning the relevance of the intestinal microbiota for the detected changes in the plasma triglyceride and cholesterol concentrations.

Our results contrast another study evaluating the role of the dietary fat supply for the intestinal microbiota of cats [10]. In this study, the diets differed in the starch source (corn starch vs. pea starch) and in the fat concentration (11% vs. 18%). Both dietary factors influenced the composition of the fecal microbiota of the cats after feeding the diets for 28 days [10]. For a data comparison with the present results, it should be considered that the dietary fat concentrations were slightly higher, and the feeding periods slightly longer in the study of Mo et al. [10]. Those factors might have contributed to the divergent results, as well as further differences in the diet composition or an impact of animal-related or external factors that varied between the studies.

As a limitation of the present investigation, it should be discussed that graduated dietary fat concentrations could be evaluated, but that the daily supplementation dose was restricted to a maximum of 1 g oil or lard per kg body weight of the cats for palatability reasons. It would have been an alternative study approach to use a moderate- or high-fat basal diet, where the addition of 0.5–1 g oil/lard per kg body weight of the animals had resulted in higher overall fat intakes of the cats. However, higher native fat concentrations as a basal treatment could have changed the baseline condition of the gut microbiome and potentially also masked specific or dose-dependent effects of the fat supplements. Nevertheless, future studies should evaluate the impact of higher fat intakes on the intestinal microbiota of cats, which would also allow for a comprehensive interpretation of available study data.

Further limitations of this study include the relatively short feeding periods as well as the absence of wash-out periods in between. A wash-out period was considered dispensable due to the chosen study design, since a modified Latin square design was used, and the same oil/fat was not supplemented in the two consecutive feeding periods to avoid any impact on the evaluated dose effects [21]. A feeding period of 21 days was expected to be long enough to detect diet-specific effects on the intestinal microbiota, as data in dogs could demonstrate that the gut microbiome adapts very quickly to different diets [47]. In this study, a stabilization of the fecal bacterial composition was observed after 6 days, and of microbial metabolites in the feces even after 2 days [47]. However, respective study data in cats are missing. It remains unclear at this stage if the observed effects and tendencies of the microbiota modulation by the dietary fat supplementation can also be detected for the long-term dietary treatment. This aspect should be evaluated in future studies.

## 5. Conclusions

The fecal microbiota of healthy adult cats was only slightly affected by variations in the dietary fat concentration and fatty acid pattern. Thus, no adverse effects on a stabilized bacterial community can be expected by moderate modifications of the dietary fat supply in felines. The effects of a longer-term treatment and higher fat intakes should be subject of future investigations.

## Figures and Tables

**Table 1:** Dry matter and nutrient concentrations of the basal diet ^1,2^ [21].

Component	Analyzed
Dry matter (g/100 g)	93.7
	In g/100 g dry matter
Crude protein	35.0
Crude fat	9.07
Crude fiber	2.24
Crude ash	11.1
Calcium	1.89
Phosphorus	1.25
Sodium	0.69
Potassium	0.97
Magnesium	0.11
	In mg/100 g dry matter
Copper	2.13
Zinc	16.9
Iron	80.6
Manganese	4.90

^1^ Analyzed by an accredited external laboratory (AGROLAB LUFA GmbH, Kiel, Germany), using the official methods for feed analyses (Commission Regulation (EC) No 152/2009 III, A, M, C, I: 2009-01, H, Procedure B: 2009-01; DIN EN 15621: 2017-10). ^2^ Composition, as specified by the manufacturer, was as follows: meat and animal by-products (duck meat meal), vegetables (dried sweet potato), plant by-products (dried tapioca, cellulose), oils and fats, flaxseed, minerals, chicory as a source of inulin (0.5%).

**Table 2:** Fatty acid composition of the basal diet, sunflower oil, fish oil and lard ^1^ [21].

	Basal Diet	Basal Diet	Sunflower Oil	Fish Oil	Lard
	mg/kg Diet ^2^	% of Total Fatty Acids ^3^			
Analyzed					
Caprylic acid C 8:0	<50.0	<0.1	<0.1	<0.1	<0.1
Capric acid C 10:0	111	0.2	<0.1	<0.1	<0.1
Lauric acid C 12:0	466	0.7	<0.1	<0.1	<0.1
Myristic acid C 14:0	478	0.7	<0.1	4.5	1.5
Myristoleic acid C 14:1	78.6	0.1	<0.1	0.1	<0.1
Pentadecanoic acid C 15:0	70.6	0.1	<0.1	0.3	<0.1
Palmitic acid C 16:0	13,100	19.6	6.2	13.5	26.9
Hexadecanoic acid trans-isomers C 16:1 trans	<50.0	<0.1	<0.1	<0.1	<0.1
Palmitoleic acid C 16:1	2310	3.4	0.1	5.7	1.8
Hexadecadienoic acid C16:2 (n-4)	<50.0	<0.1	<0.1	0.7	<0.1
Hexadecatrienoic acid C16:3 (n-3)	<50.0	<0.1	<0.1	<0.1	<0.1
Margaric acid C 17:0	178	0.3	<0.1	0.4	0.3
Heptadecenoic acid C 17:1	<50.0	<0.1	<0.1	<0.1	<0.1
Stearic acid C 18:0	4440	6.6	3.6	2.6	18.2
Octadecenoic acid trans-isomers C 18:1 trans	236	0.4	<0.1	1.2	0.1
Oleic acid C 18:1	23,500	35.1	28.0	12.3	36.9
Petroselinic acid C 18:1	<50.0	<0.1	<0.1	<0.1	<0.1
cis-vaccenic acid C 18:1	1220	1.8	0.6	5.5	2.4
Octadecadienoic acid trans-isomers C 18:2 trans	122	0.2	<0.1	0.9	<0.1
Linoleic acid C 18:2 (n-6)	15,800	23.6	59.6	0.8	9.0
Octadecatrienoic acid trans-isomers C 18:3 trans	58.8	0.1	<0.1	0.2	<0.1
alpha-linolenic acid C 18:3 (n-3)	2900	4.3	<0.1	0.7	0.8
gamma-linolenic acid C 18:3 (n-6)	79.8	0.1	<0.1	0.1	<0.1
Stearidonic acid C 18:4 (n-3)			<0.1	3.5	<0.1
Octadecatetraenoic acid C 18:4 (n-3)	<50.0	<0.1			
Arachidic acid C 20:0	282	0.4	0.3	<0.1	0.2
Eicosenoic acid C 20:1	311	0.5	0.2	11.2	0.8
Eicosadienoic acid C 20:2 (n-6)	98.3	0.1	<0.1	0.2	0.4
Eicosatrienoic acid C 20:3 (n-3)	<50.0	<0.1	<0.1	<0.1	<0.1
Eicosatrienoic acid C 20:3 (n-6)	94.0	0.1	<0.1	<0.1	<0.1
Arachidonic acid C 20:4 (n-6)	552	0.8	<0.1	0.3	0.2
Eicosatetraenoic acid C20:4 (n-3)	<50.0	<0.1	<0.1	0.6	<0.1
Eicosapentaenoic acid C 20:5 (n-3)	<50.0	<0.1	<0.1	13.9	<0.1
Heneicosanoic acid C 21:0	57.6	0.1	<0.1	<0.1	<0.1
Behenic acid C 22:0	174	0.3	0.8	<0.1	<0.1
Docosenoic acid trans-isomers C 22:1 trans	<50.0	<0.1	<0.1	<0.1	<0.1
Docosenoic acid C 22:1	<50.0	<0.1	<0.1	0.7	<0.1
Cetoleic acid C 22:1	<50.0	<0.1	<0.1	10.4	<0.1
Docosadienoic acid C 22:2 (n-6)	<50.0	<0.1	<0.1	<0.1	<0.1
Docosatrienoic acid C 22:3	<50.0	<0.1	<0.1	<0.1	<0.1
Docosatetraenoic acid C 22:4 (n-6)	131	0.2	<0.1	0.2	<0.1
Docosapentaenoic acid C 22:5 (n-3)	<50.0	<0.1	<0.1	0.9	<0.1
Docosapentaenoic acid C 22:5 (n-6)	<50.0	<0.1	<0.1	<0.1	<0.1
Docosahexaenoic acid C 22:6 (n-3)	<50.0	<0.1	<0.1	7.3	<0.1
Tricosanoic acid C 23:0	<50.0	<0.1	<0.1	<0.1	<0.1
Lignoceric acid C 24:0	110	0.2	0.3	<0.1	<0.1
Nervonic acid C 24:1	<50.0	<0.1	<0.1	1.1	<0.1
Calculated					
Sum saturated fatty acids	19,500	29.1	11.2	21.3	47.1
Sum monounsaturated fatty acids	27,700	41.3	28.9	48.2	42.0
Total sum fatty acids	67,000				
Sum polyunsaturated fatty acids	19,800	29.6	59.6	30.3	10.4
Sum trans fatty acids	417	0.62	<0.1	2.3	0.1
n-3 fatty acids	2900	4.33	<0.1	26.9	0.8
n-6 fatty acids	16,800	25.1	59.6	1.6	9.6
n-9 fatty acids	23,800	35.5	28.2	25.3	37.7
n-6:n-3 fatty acids ratio	5.79:1	5.79:1	>596:1	0.06:1	12:1

^1^ Analyzed by an accredited external laboratory (AGROLAB LUFA GmbH, Kiel, Germany), using the methods of the German Society for Fat Science (DGF) (C-VI 11a: 2016 (mod.) + DGF C-VI 10a: 2016 (mod.)). ^2^ Quantitative measurements of the fatty acids in the total diet. ^3^ Relative amounts of the single fatty acids, expressed as % of the total fatty acids in the diet, oils, and lard. Values reported with a “<” symbol: below the detection limit of the analyzing laboratory.

**Table 3:** Feed intake and body weight (BW) of cats fed a basal diet without (“w/o”) or with the supplementation of sunflower oil (0.5 g or 1 g/kg body weight/day), fish oil (0.5 g or 1 g/kg body weight/day) or lard (0.5 g or 1 g/kg body weight/day). Means and pooled standard error of the means (SEM) [21].

	w/o	Sunflower Oil		Fish Oil		Lard		SEM	*p* Value for the Overall Treatment		
									Sunflower Oil	Fish Oil	Lard
		0.5 g	1.0 g	0.5 g	1.0 g	0.5 g	1.0 g				
	n = 9	n = 10	n = 10	n = 10	n = 10	n = 9	n = 8				
Per Feeding Period											
Feed intake (g/day)	66.0	64.2	61.0	60.2	60.9	63.0	60.5	1.07	0.1348	0.0808	0.3153
Feed intake (g/kg BW/day)	13.8	13.3	12.5	12.3	12.6	13.0	13.0	0.30	0.1226	0.0952	0.2218
BW (kg)	4.92	4.97	4.96	4.97	4.97	4.92	4.73	0.10	0.9784	0.9885	0.4541
For the Collection Period											
Feed intake (g/day)	63.6	60.6	59.4	58.0	54.8	61.0	57.5	1.30	0.2861	0.0675	0.1115
Feed intake (g/kg BW/day)	13.2	12.6	12.2	11.9	11.4	12.6	12.3	0.35	0.2774	0.0733	0.1044
BW (kg)	4.92	4.98	4.96	4.97	4.98	4.95	4.73	0.10	0.9697	0.8897	0.2511

**Table 4:** Fecal pH and bacterial metabolites in the feces of cats fed a basal diet without (“w/o”) or with the supplementation of sunflower oil (0.5 g or 1 g/kg body weight/day), fish oil (0.5 g or 1 g/kg body weight/day) or lard (0.5 g or 1 g/kg body weight/day). Means and pooled standard error of the means (SEM).

	w/o	Sunflower Oil		Fish Oil		Lard		SEM	*p* Value for the Overall Treatment ^1^		
		0.5 g	1.0 g	0.5 g	1.0 g	0.5 g	1.0 g		Sunflower Oil	Fish Oil	Lard
	n = 9	n = 10	n = 10	n = 10	n = 10	n = 9	n = 8				
pH	6.06	6.17	6.25	6.10	6.08	6.40	6.27	0.06	0.5126	0.9509	0.6658
µmol/g											
D-lactate	0.15	0.11	0.14	1.00	0.15	0.16	0.19	0.10	0.4449	0.4254	0.8777
L-lactate	0.03	0.01	0.03	0.86	0.01	0.02	0.02	0.11	0.1830	0.3508	0.6850
Ammonium	41.1	36.2	39.6	35.6	38.9	45.4	36.5	1.45	0.3786	0.6614	0.0837
Acetic acid	95.3	80.6	95.7	96.6	115	107	99.5	4.06	0.6865	0.1263	0.2844
Propionic acid	31.6	30.4	38.9	36.2	44.2	43.3	38.5	2.36	0.5033	0.0603	0.0884
i-butyric acid	3.45 ^ab^	2.95	2.99	2.78	3.26	2.80 ^a^	3.75 ^b^	0.13	0.4439	0.0431	0.0332
n-butyric acid	25.8	18.2	25.7	25.4	35.5	31.5	19.6	2.24	0.3567	0.5706	0.1471
i-valeric acid	5.84	5.16	5.18	5.30	5.51	4.62	5.95	0.21	0.5638	0.4563	0.1469
n-valeric acid	11.9	10.6	14.5	13.8	14.4	15.6	12.2	0.66	0.2364	0.1754	0.0946
Total short-chain fatty acids	174	148	183	180	218	204	179	8.11	0.6036	0.1149	0.1903
Mol.%											
Acetic acid	56.0	54.6	52.3	54.3	52.8	53.1	55.7	0.74	0.5741	0.4877	0.3710
Propionic acid	18.1	19.2	20.0	19.6	20.1	20.7	21.2	0.50	0.4622	0.1262	0.0952
i-butyric acid	2.03	2.17	1.85	1.72	1.64	1.51	2.12	0.09	0.6649	0.3000	0.0437
n-butyric acid	13.8	12.6	14.1	13.2	15.8	14.4	10.8	0.75	0.4999	0.5930	0.2236
i-valeric acid	3.49	3.80	3.41	3.32	2.77	2.54	3.37	0.17	0.7996	0.2667	0.0951
n-valeric acid	6.56	7.69	8.35	7.78	6.84	7.73	6.79	0.25	0.2201	0.3167	0.3259

^1^ Pairwise comparisons were separately evaluated for the 3 dietary treatments (sunflower oil, fish oil, lard) (see Section 2.4). Different superscript letters in the same row indicate a significant group difference (*p* < 0.05).

**Table 5:** Concentrations of biogenic amines ^1^ in the feces of cats fed a basal diet without (“w/o”) or with the supplementation of sunflower oil (0.5 g or 1 g/kg body weight/day), fish oil (0.5 g or 1 g/kg body weight/day) or lard (0.5 g or 1 g/kg body weight/day). Means and pooled standard error of the means (SEM).

	w/o	Sunflower Oil		Fish Oil		Lard		SEM	*p* Value for the Overall Treatment		
		0.5 g	1.0 g	0.5 g	1.0 g	0.5 g	1.0 g		Sunflower Oil	Fish Oil	Lard
	n = 9	n = 10	n = 10	n = 10	n = 10	n = 9	n = 8				
µmol/g											
Putrescine	6.58	5.86	6.02	6.31	7.03	5.09	7.40	0.63	0.9210	0.9069	0.3303
Histamine	0.61	0.75	0.70	1.03	0.86	0.63	0.99	0.08	0.8554	0.4619	0.1909
Cadaverine	18.6	20.0	19.7	19.4	20.8	15.6	19.8	1.23	0.3662	0.6178	0.4321
Spermidine	0.72	0.71	0.65	0.66	0.67	0.65	0.75	0.03	0.8433	0.7451	0.6383
Tyramine	0.23	0.19	0.26	0.35	0.28	0.12	0.24	0.05	0.2044	0.7436	0.0659
Spermine	0.06	0.04	0.04	0.05	0.05	0.04	0.05	0.00	0.3040	0.7056	0.1370
Total biogenic amines	26.8	27.5	27.4	27.8	29.7	22.1	29.2	1.77	0.6904	0.5416	0.3770

^1^ Propylamine and phenylethylamine: below the detection limit in all samples.

**Table 6:** Alpha diversity of the fecal microbiota of cats fed a basal diet without (“w/o”) or with the supplementation of sunflower oil (0.5 g or 1 g/kg body weight/day), fish oil (0.5 g or 1 g/kg body weight/day), or lard (0.5 g or 1 g/kg body weight/day). Means and pooled standard error of the means (SEM).

	w/o	Sunflower Oil		Fish Oil		Lard		SEM	*p* Value for the Overall Treatment		
		0.5 g	1.0 g	0.5 g	1.0 g	0.5 g	1.0 g		Sunflower Oil	Fish Oil	Lard
	n = 9	n = 10	n = 10	n = 10	n = 10	n = 9	n = 8				
Richness	112	112	109	113	111	110	120	2.49	0.8733	0.9515	0.3574
Shannon Index	3.27	3.30	3.29	3.37	3.32	3.30	3.50	0.07	0.9460	0.8205	0.3555
Evenness	0.69	0.70	0.70	0.71	0.70	0.70	0.73	0.01	0.8776	0.7896	0.4088

**Table 7:** Relative abundance (%) of the dominant bacterial phyla in the feces of cats fed a basal diet without (“w/o”) or with the supplementation of sunflower oil (0.5 g or 1 g/kg body weight/day), fish oil (0.5 g or 1 g/kg body weight/day) or lard (0.5 g or 1 g/kg body weight/day). Means and pooled standard error of the means (SEM). In brackets: n (samples with the detected phylum).

	w/o	Sunflower Oil		Fish Oil		Lard		SEM	*p* Value for the Overall Treatment		
		0.5 g	1.0 g	0.5 g	1.0 g	0.5 g	1.0 g		Sunflower Oil	Fish Oil	Lard
*Actinobacteria*	39.5 (n = 9)	37.3 (n = 10)	40.0 (n = 10)	36.7 (n = 10)	39.7 (n = 10)	40.5 (n = 9)	36.2 (n = 8)	2.31	0.8099	0.3135	0.5409
*Bacteroidetes*	13.0 (n = 9)	15.4 (n = 10)	13.8 (n = 10)	14.3 (n = 10)	14.9 (n = 10)	13.1 (n = 8)	13.3 (n = 8)	1.27	0.8979	0.8116	0.9566
*Epsilonbacteraeota*	0.09 (n = 6)	0.07 (n = 6)	0.15 (n = 5)	0.10 (n = 8)	0.09 (n = 6)	0.07 (n = 5)	0.11 (n = 7)	0.01	*	*	0.1810
*Firmicutes*	46.5 (n = 9)	44.9 (n = 10)	44.8 (n = 10)	46.7 (n = 10)	42.7 (n = 10)	46.2 (n = 9)	49.2 (n = 8)	2.09	0.9987	0.5379	0.4870
*Fusobacteria*	0.31 (n = 6)	0.31 (n = 4)	0.15 (n = 3)	0.19 (n = 5)	0.66 (n = 5)	0.30 (n = 3)	0.11 (n = 6)	0.09	*	*	*
*Proteobacteria*	0.89 (n = 7)	2.07 (n = 10)	1.18 (n = 10)	2.42 (n = 9)	2.45 (n = 9)	1.66 (n = 8)	1.04 (n = 8)	0.38	0.6205	0.2217	0.6617

* *p* value could not be calculated.

**Table 8:** Relative abundance (%) of the dominant bacterial genera in the feces of cats fed a basal diet without (“w/o”) or with the supplementation of sunflower oil (0.5 g or 1 g/kg body weight/day), fish oil (0.5 g or 1 g/kg body weight/day) or lard (0.5 g or 1 g/kg body weight/day). Means and pooled standard error of the means (SEM). In brackets: n (samples with the detected genus).

	w/o	Sunflower Oil		Fish Oil		Lard		SEM	*p* Value for the Overall Treatment		
		0.5 g	1.0 g	0.5 g	1.0 g	0.5 g	1.0 g		Sunflower Oil	Fish Oil	Lard
*Alloprevotella*	2.12 (n = 7)	0.65 (n = 10)	0.63 (n = 9)	0.82 (n = 8)	1.83 (n = 9)	1.47 (n = 8)	1.55 (n = 7)	0.22	0.3876	0.3759	0.8157
*Anaerobiospirillum*	0.21 (n = 6)	0.22 (n = 8)	0.20 (n = 5)	0.16 (n = 8)	1.71 (n = 6)	0.46 (n = 6)	0.13 (n = 8)	0.19	*	*	0.3415
*Bacteroides*	2.32 (n = 7)	1.07 (n = 9)	2.57 (n = 7)	1.57 (n = 8)	1.06 (n = 10)	1.04 (n = 8)	1.14 (n = 7)	0.23	0.1658	0.4853	0.6377
*Bifidobacterium*	20.3 (n = 9)	22.1 (n = 10)	21.1 (n = 10)	17.2 (n = 10)	23.0 (n = 10)	22.4 (n = 9)	17.2 (n = 8)	1.95	0.9863	0.2322	0.2298
*Blautia*	11.8 (n = 9)	9.75 (n = 10)	10.2 (n = 10)	11.3 (n = 10)	9.37 (n = 10)	10.3 (n = 9)	11.7 (n = 8)	0.77	0.4971	0.3867	0.3822
*Catenibacterium*	3.71 (n = 9)	4.31 (n = 10)	5.92 (n = 10)	7.13 (n = 9)	2.97 (n = 10)	6.24 (n = 9)	6.97 (n = 8)	1.11	0.0734	0.4288	0.2163
*Catenisphaera*	0.60 (n = 8)	1.25 (n = 9)	0.26 (n = 10)	0.13 (n = 7)	0.28 (n = 8)	0.10 (n = 9)	0.39 (n = 6)	0.18	0.6360	0.2446	0.2450
*Collinsella*	18.2 (n = 9)	14.4 (n = 10)	17.9 (n = 10)	18.6 (n = 10)	15.9 (n = 10)	17.2 (n = 9)	18.3 (n = 8)	1.02	0.1793	0.6083	0.6267
*Faecalibacterium*	0.60 (n = 8)	0.52 (n = 8)	0.41 (n = 9)	0.44 (n = 10)	0.48 (n = 10)	0.29 (n = 6)	0.26 (n = 8)	0.07	0.5796	0.5601	0.4189
*Helicobacter*	0.08 (n = 6)	0.07 (n = 6)	0.15 (n = 5)	0.09 (n = 8)	0.09 (n = 6)	0.06 (n = 5)	0.10 (n = 7)	0.01	*	*	0.1160
*Holdemanella*	6.86 (n = 9)	5.56 (n = 10)	7.28 (n = 10)	6.35 (n = 10)	5.21 (n = 10)	7.14 (n = 9)	4.29 (n = 8)	0.87	0.3157	0.5714	0.3187
*Lachnoclostridium*	2.90 (n = 9)	2.55 (n = 10)	2.58 (n = 10)	2.40 (n = 10)	2.71 (n = 10)	2.28 (n = 9)	3.00 (n = 8)	0.20	0.4613	0.2644	0.2619
*Lachnospiraceae* NK4A136 group	0.22 (n = 7)	0.14 (n = 8)	0.14 (n = 5)	0.16 (n = 8)	0.21 (n = 10)	0.18 (n = 8)	0.15 (n = 8)	0.02	*	0.6150	0.8127
*Libanicoccus*	0.27 (n = 8)	0.20 (n = 8)	0.30 (n = 9)	0.28 (n = 10)	0.38 (n = 8)	0.24 (n = 8)	0.22 (n = 8)	0.03	0.1505	0.3261	0.8177
*Megasphaera*	3.99 (n = 7)	4.83 (n = 9)	3.52 (n = 9)	3.79 (n = 10)	5.50 (n = 9)	4.57 (n = 9)	4.12 (n = 8)	0.49	0.2721	0.4440	0.8564
*Negativibacillus*	0.70 (n = 9)	0.68 (n = 10)	0.65 (n = 10)	0.70 (n = 10)	0.74 (n = 10)	0.74 (n = 8)	0.82 (n = 8)	0.06	0.9934	0.9247	0.1571
*Parabacteroides*	0.18 (n = 6)	0.13 (n = 7)	0.11 (n = 7)	0.10 (n = 4)	0.12 (n = 7)	0.06 (n = 6)	0.07 (n = 7)	0.02	*	*	0.4080
*Peptoclostridium*	3.77 (n = 9)	4.04 (n = 10)	3.99 (n = 10)	4.22 (n = 10)	2.97 (n = 10)	3.43 (n = 9)	4.78 (n = 8)	0.32	0.9831	0.1320	0.0887
*Peptococcus*	0.34 (n = 9)	0.25 (n = 10)	0.27 (n = 10)	0.33 (n = 10)	0.26 (n = 10)	0.28 (n = 9)	0.31 (n = 8)	0.04	0.2825	0.7653	0.7575
*Prevotella* 9	9.33 (n = 9)	13.6 (n = 10)	11.2 (n = 10)	13.6 (n = 9)	12.0 (n = 10)	10.4 (n = 8)	10.8 (n = 8)	1.20	0.7030	0.6922	0.9414
*Ruminiclostridium* 9	0.09 (n = 7)	0.14 (n = 7)	0.12 (n = 7)	0.17 (n = 7)	0.10 (n = 9)	0.09 (n = 6)	0.09 (n = 6)	0.01	0.0558	0.1771	*
*Ruminococcaceae* UCG-004	0.10 (n = 7)	0.11 (n = 7)	0.13 (n = 7)	0.10 (n = 6)	0.10 (n = 7)	0.08 (n = 7)	0.16 (n = 4)	0.01	0.5216	*	0.1034
*Sellimonas*	0.77 (n = 9)	0.70 (n = 10)	0.73 (n = 10)	0.83 (n = 10)	0.78 (n = 10)	0.81 (n = 9)	0.62 (n = 8)	0.05	0.5188	0.9174	0.4700
*Slackia*	0.14 (n = 9)	0.15 (n = 9)	0.19 (n = 8)	0.12 (n = 10)	0.12 (n = 10)	0.10 (n = 8)	0.15 (n = 7)	0.01	0.4383	0.6975	0.0994
*Solobacterium*	1.37 (n = 9)	1.81 (n = 10)	1.16 (n = 10)	1.46 (n = 10)	1.44 (n = 10)	1.24 (n = 9)	0.94 (n = 8)	0.16	0.3693	0.9485	0.0470
*Subdoligranulum*	2.95 (n = 9)	3.52 (n = 10)	1.95 (n = 10)	2.52 (n = 10)	3.04 (n = 10)	2.62 (n = 9)	3.65 (n = 7)	0.34	0.1791	0.8736	0.7029
unknown (Family *Atopobiaceae*)	0.12 (n = 6)	0.09 (n = 6)	0.11 (n = 8)	0.08 (n = 7)	0.09 (n = 7)	0.11 (n = 7)	0.12 (n = 5)	0.01	*	*	0.3230
unknown (Family *Bifidobacteriaceae*)	0.20 (n = 7)	0.19 (n = 9)	0.23 (n = 9)	0.18 (n = 8)	0.24 (n = 8)	0.25 (n = 8)	0.21 (n = 5)	0.02	0.4716	0.1101	0.0045
unknown (Family *Eggerthellaceae*)	0.22 (n = 9)	0.25 (n = 9)	0.24 (n = 9)	0.20 (n = 10)	0.20 (n = 9)	0.18 (n = 9)	0.17 (n = 8)	0.02	0.7036	0.9194	0.4209
unknown (Family *Erysipelotrichaceae*)	0.15 (n = 8)	0.16 (n = 6)	0.19 (n = 7)	0.20 (n = 7)	0.11 (n = 8)	0.20 (n = 7)	0.14 (n = 7)	0.02	0.0844	0.7029	0.9985
unknown (Family Family XIII)	0.43 (n = 7)	0.32 (n = 9)	0.27 (n = 7)	0.20 (n = 8)	0.17 (n = 7)	0.16 (n = 7)	0.39 (n = 5)	0.04	0.4242	0.0169	0.2041
unknown (Family *Lachnospiraceae*)	3.59 (n = 9)	2.85 (n = 10)	2.97 (n = 10)	2.82 (n = 10)	2.75 (n = 10)	2.77 (n = 9)	3.40 (n = 8)	0.24	0.1716	0.3801	0.4599
unknown (Family *Ruminococcaceae*)	0.07 (n = 6)	0.10 (n = 5)	0.09 (n = 7)	0.09 (n = 7)	0.04 (n = 3)	0.05 (n = 7)	0.07 (n = 7)	0.01	*	*	0.5895

* *p* value could not be calculated.

## Data Availability

Sequencing data are available at: http://www.ncbi.nlm.nih.gov/bioproject/1219520, accessed on 19 February 2025. Further raw data supporting the conclusions of this article will be made available by the authors on request.

## Supplementary Materials

The following supporting information can be downloaded at: https://www.mdpi.com/article/10.3390/metabo15040215/s1, Table S1: Relative abundance (%) of bacterial genera in the feces of cats fed a basal diet without (“w/o”) or with the supplementation of sunflower oil (0.5 g or 1 g/kg body weight/day), fish oil (0.5 g or 1 g/kg body weight/day) or lard (0.5 g or 1 g/kg body weight/day). Means and pooled standard error of the means (SEM). In brackets: n (samples with the detected genus). Table S2: Spearman’s rho correlation coefficient (*p*-value) when correlating the 3 doses of the dietary sunflower treatment (0 g, 0.5 g and 1 g/kg body weight/day), fish oil treatment (0 g, 0.5 g and 1 g/kg body weight/day) and lard treatment (0 g, 0.5 g and 1 g/kg body weight/day) with the relative abundance (%) of bacterial phyla in the feces of cats. Table S3: Spearman’s rho correlation coefficient (*p*-value) when correlating the 3 doses of the dietary sunflower treatment (0 g, 0.5 g and 1 g/kg body weight/day), fish oil treatment (0 g, 0.5 g and 1 g/kg body weight/day) and lard treatment (0 g, 0.5 g and 1 g/kg body weight/day) with the relative abundance (%) of bacterial genera in the feces of cats.
