# Supplementary material for: Impact of the Dietary Fat Concentration and Source on the Fecal Microbiota of Healthy Adult Cats
Source: Metabolites. 2025 Mar 22;15(4):215. doi: 10.3390/metabo15040215 (PMC12028789; doi:10.3390/metabo15040215)
Supplement: Supplementary file 1 [file metabolites-15-00215-s001.zip › Table S1_REVISED_np220325.pdf]

Table S1: Relative abundance (%) of bacterial genera in the feces of cats fed a basal diet without ("w/o") or with the supplementation of sunflower oil (0.5 g or 1 g/kg body weight/day), fish oil (0.5 g or 1 g/kg body weight/day) or lard (0.5 g or 1 g/kg body weight/day). Means and pooled standard error of the means (SEM). In brackets: n (samples with the detected genus).

	w/o	Sunflower oil		Fish oil		Lard		SEM
		**0.5 g**	**1.0 g**	**0.5 g**	**1.0 g**	**0.5 g**	**1.0 g**	
Acidaminococcus	0.46 (n=4)	0.14 (n=5)	0.36 (n=4)	0.16 (n=6)	0.12 (n=6)	0.26 (n=5)	0.20 (n=4)	0.05
Allisonella	0.06 (n=6)	0.05 (n=8)	0.09 (n=4)	0.07 (n=5)	0.06 (n=8)	0.07 (n=6)	0.08 (n=5)	0.01
Anaerofilum	0.11 (n=2)	0.17 (n=1)	0.08 (n=2)	0.07 (n=1)	0.11 (n=4)	0.12 (n=2)	0.13 (n=1)	0.02
Asaccharospora	0.03 (n=2)	0.09 (n=1)	0.08 (n=2)	0.05 (n=2)	0.06 (n=3)	0.05 (n=2)	- (n=0)	0.01
Anaerostipes	0.19 (n=2)	0.05 (n=2)	- (n=0)	- (n=0)	0.04 (n=2)	0.02 (n=1)	0.03 (n=1)	0.04
Butyricicoccus	0.06 (n=5)	0.04 (n=4)	0.04 (n=6)	0.03 (n=4)	0.07 (n=3)	0.03 (n=8)	0.05 (n=6)	0.00
Campylobacter	0.07 (n=1)	0.02 (n=2)	0.03 (n=1)	0.08 (n=1)	0.02 (n=1)	0.02 (n=1)	0.02 (n=4)	0.01
***Christensenellaceae* R-7 group**	0.03 (n=2)	0.04 (n=4)	0.03 (n=4)	0.03 (n=1)	0.02 (n=2)	0.04 (n=2)	0.05 (n=2)	0.01
***Clostridium sensu stricto* 1**	0.72 (n=3)	0.05 (n=1)	0.16 (n=2)	1.07 (n=3)	0.46 (n=4)	0.05 (n=2)	0.12 (n=3)	0.20
***Defluviitaleaceae* UCG-011**	0.02 (n=1)	0.03 (n=5)	0.05 (n=1)	- (n=0)	0.02 (n=2)	0.04 (n=1)	0.03 (n=2)	0.00
Denitrobacterium	0.03 (n=1)	0.05 (n=3)	0.04 (n=2)	0.03 (n=2)	0.04 (n=1)	0.04 (n=1)	0.03 (n=4)	0.00
Desulfovibrio	0.17 (n=4)	0.08 (n=5)	0.17 (n=6)	0.17 (n=3)	0.18 (n=5)	0.11 (n=3)	0.12 (n=5)	0.02
Dialister	0.53 (n=3)	0.83 (n=3)	0.48 (n=4)	0.63 (n=5)	0.76 (n=4)	0.79 (n=4)	0.91 (n=4)	0.05
**DTU089**	0.02	0.03	0.02	0.02	0.01	-	-	0.00

	(n=1)	(n=4)	(n=3)	(n=1)	(n=1)	(n=0)	(n=0)	
Eisenbergiella	0.14 (n=1)	0.09 (n=2)	0.05 (n=1)	0.05 (n=2)	0.12 (n=1)	0.03 (n=2)	- (n=0)	0.01
Enorma	0.09 (n=5)	0.07 (n=7)	0.09 (n=5)	0.07 (n=6)	0.08 (n=4)	0.12 (n=4)	0.06 (n=4)	0.01
Flavonifractor	0.04 (n=2)	0.06 (n=1)	0.03 (n=3)	0.03 (n=3)	0.04 (n=3)	0.02 (n=3)	- (n=0)	0.00
Fournierella	0.02 (n=2)	0.05 (n=2)	0.04 (n=1)	0.04 (n=2)	0.03 (n=2)	0.04 (n=2)	0.05 (n=3)	0.00
Fusobacterium	0.31 (n=6)	0.31 (n=4)	0.15 (n=3)	0.19 (n=5)	0.66 (n=5)	0.30 (n=3)	0.11 (n=6)	0.09
Intestinimonas	0.02 (n=3)	0.03 (n=2)	0.05 (n=2)	0.03 (n=1)	0.03 (n=2)	- (n=0)	0.05 (n=1)	0.01
Lachnospira	0.58 (n=6)	1.16 (n=5)	0.29 (n=7)	0.36 (n=5)	0.43 (n=6)	0.17 (n=5)	0.31 (n=5)	0.09
***Lachnospiraceae* UCG-010**	0.02 (n=2)	0.01 (n=1)	0.03 (n=3)	- (n=0)	0.03 (n=1)	- (n=0)	0.03 (n=1)	0.00
Marvinbryantia	0.19 (n=3)	0.15 (n=4)	0.06 (n=3)	0.02 (n=2)	0.06 (n=3)	0.09 (n=3)	0.05 (n=4)	0.02
Megamonas	0.88 (n=3)	0.34 (n=6)	0.96 (n=6)	0.49 (n=7)	2.81 (n=7)	1.83 (n=6)	0.76 (n=4)	0.41
Mogibacterium	0.19 (n=2)	0.06 (n=2)	0.04 (n=2)	0.06 (n=4)	0.04 (n=3)	0.03 (n=3)	0.01 (n=1)	0.01
Odoribacter	0.07 (n=6)	0.07 (n=4)	0.12 (n=3)	0.10 (n=3)	0.05 (n=4)	0.09 (n=3)	0.04 (n=3)	0.01
Oscillibacter	0.06 (n=5)	0.05 (n=5)	0.05 (n=4)	0.04 (n=6)	0.06 (n=4)	0.05 (n=3)	0.04 (n=4)	0.01
Oscillospira	0.04 (n=2)	0.06 (n=3)	0.05 (n=2)	0.03 (n=1)	0.04 (n=2)	0.09 (n=1)	0.08 (n=1)	0.01
Phascolarctobacterium	0.30 (n=5)	0.20 (n=6)	0.14 (n=4)	0.22 (n=4)	0.36 (n=5)	0.30 (n=4)	0.09 (n=4)	0.05
Phocea	0.05	0.02	-	0.02	0.04	0.02	0.03	0.01

	(n=1)	(n=2)	(n=0)	(n=1)	(n=2)	(n=1)	(n=1)	
Pygmaiobacter	0.06 (n=5)	0.05 (n=6)	0.07 (n=3)	0.05 (n=2)	0.07 (n=1)	0.06 (n=3)	0.07 (n=2)	0.01
Romboutsia	0.72 (n=4)	0.80 (n=3)	1.24 (n=6)	0.74 (n=5)	1.30 (n=4)	0.34 (n=5)	2.38 (n=5)	0.33
Roseburia	0.16 (n=6)	0.17 (n=4)	0.22 (n=5)	0.16 (n=4)	0.12 (n=6)	0.13 (n=4)	0.29 (n=4)	0.03
Ruminiclostridium	- (n=0)	0.03 (n=2)	0.02 (n=1)	0.02 (n=2)	0.04 (n=1)	0.02 (n=1)	0.03 (n=1)	0.00
Ruminiclostridium 5	0.05 (n=5)	0.06 (n=5)	0.05 (n=5)	0.04 (n=6)	0.04 (n=5)	0.03 (n=3)	0.07 (n=3)	0.00
***Ruminococcaceae* UCG-005**	0.29 (n=1)	0.15 (n=2)	0.01 (n=1)	- (n=0)	0.08 (n=1)	0.04 (n=1)	0.04 (n=2)	0.04
***Ruminococcaceae* UCG-009**	0.12 (n=5)	0.20 (n=6)	0.19 (n=5)	0.10 (n=7)	0.19 (n=6)	0.20 (n=4)	0.14 (n=5)	0.02
***Ruminococcaceae* UCG-014**	0.20 (n=6)	0.16 (n=9)	0.16 (n=5)	0.13 (n=4)	0.10 (n=5)	0.22 (n=4)	0.11 (n=6)	0.03
Succinivibrio	0.57 (n=6)	2.47 (n=7)	1.40 (n=6)	3.15 (n=6)	1.27 (n=7)	1.61 (n=6)	1.45 (n=4)	0.47
Sutterella	0.14 (n=3)	0.09 (n=6)	0.07 (n=3)	0.06 (n=7)	0.18 (n=6)	0.07 (n=4)	0.10 (n=6)	0.02
Turicibacter	0.02 (n=2)	- (n=0)	0.03 (n=1)	0.02 (n=1)	0.68 (n=2)	0.72 (n=3)	0.02 (n=1)	0.17
Tyzzerella	0.25 (n=3)	0.07 (n=2)	0.03 (n=2)	- (n=0)	0.08 (n=1)	- (n=0)	0.04 (n=1)	0.05
Tyzzerella 4	0.06 (n=2)	0.22 (n=2)	0.34 (n=3)	0.19 (n=1)	0.02 (n=1)	0.11 (n=2)	0.09 (n=1)	0.06
**unknown (Family *Coriobacteriaceae*)**	0.07 (n=3)	- (n=0)	0.13 (n=2)	0.08 (n=3)	0.07 (n=1)	0.06 (n=5)	0.05 (n=3)	0.01
**unknown (Family *Muribaculaceae*)**	0.05 (n=5)	0.07 (n=5)	0.05 (n=5)	0.03 (n=5)	0.07 (n=4)	0.04 (n=2)	0.07 (n=3)	0.01
**unknown (Family *Peptostreptococcaceae*)**	0.05	0.04	0.09	0.07	0.04	0.09	0.75	0.15

	(n=3)	(n=2)	(n=4)	(n=3)	(n=3)	(n=2)	(n=5)	
**unknown (Family *Prevotellaceae*)**	0.09<br>(n=5)	0.11<br>(n=7)	0.15<br>(n=6)	0.08<br>(n=7)	0.11<br>(n=6)	0.12<br>(n=4)	0.08<br>(n=7)	0.01
**unknown (Family *Succinivibrionaceae*)**	0.25<br>(n=2)	0.13<br>(n=5)	0.16<br>(n=5)	0.13<br>(n=5)	0.11<br>(n=6)	0.10<br>(n=3)	0.09<br>(n=4)	0.02
**unknown (Kingdom Bacteria)**	0.08<br>(n=3)	0.07<br>(n=2)	0.07<br>(n=4)	0.05<br>(n=1)	0.08<br>(n=2)	0.08<br>(n=2)	0.11<br>(n=2)	0.01
**unknown (Order *Coriobacteriales*)**	0.04<br>(n=2)	0.03<br>(n=3)	0.05<br>(n=1)	0.02<br>(n=4)	0.04<br>(n=2)	0.03<br>(n=4)	0.02<br>(n=2)	0.00
**unknown (Phylum *Firmicutes*)**	0.06<br>(n=4)	0.04<br>(n=4)	0.06<br>(n=2)	-<br>(n=0)	0.08<br>(n=3)	0.06<br>(n=4)	0.08<br>(n=2)	0.01
