# Supplementary material for: Impact of the Dietary Fat Concentration and Source on the Fecal Microbiota of Healthy Adult Cats
Source: Metabolites. 2025 Mar 22;15(4):215. doi: 10.3390/metabo15040215 (PMC12028789; doi:10.3390/metabo15040215)
Supplement: Supplementary file 1 [file metabolites-15-00215-s001.zip › Table S2_correlation phyla_np220325.pdf]

Table S2: Spearman's rho correlation coefficient (*P*-value) when correlating the 3 doses of the dietary sunflower treatment (0 g, 0.5 g and 1 g/kg body weight/day), fish oil treatment (0 g, 0.5 g and 1 g/kg body weight/day) and lard treatment (0 g, 0.5 g and 1 g/kg body weight/day) with the relative abundance (%) of bacterial phyla in the feces of cats.

	**Sunflower oil**	**Fish oil**	**Lard**
Actinobacteria	0.027 (0.891)	-0.030 (0.877)	-0.041 (0.841)
Bacteroidetes	0.055 (0.776)	0.081 (0.675)	0.011 (0.959)
Epsilonbacteraeota	0.431 (0.084)	0.157 (0.509)	0.042 (0.869)
Firmicutes	0.011 (0.956)	-0.021 (0.915)	0.039 (0.849)
Fusobacteria	0.239 (0.431)	0.220 (0.413)	-0.233 (0.403)
Proteobacteria	-0.066 (0.742)	0.073 (0.730)	0.109 (0.622)
