# Supplementary material for: Impact of the Dietary Fat Concentration and Source on the Fecal Microbiota of Healthy Adult Cats
Source: Metabolites. 2025 Mar 22;15(4):215. doi: 10.3390/metabo15040215 (PMC12028789; doi:10.3390/metabo15040215)
Supplement: Supplementary file 1 [file metabolites-15-00215-s001.zip › Table S3_correlation genera_np220325.pdf]

Table S3: Spearman's rho correlation coefficient (*P*-value) when correlating the 3 doses of the dietary sunflower treatment (0 g, 0.5 g and 1 g/kg body weight/day), fish oil treatment (0 g, 0.5 g and 1 g/kg body weight/day) and lard treatment (0 g, 0.5 g and 1 g/kg body weight/day) with the relative abundance (%) of bacterial genera in the feces of cats.

	**Sunflower oil**	**Fish oil**	**Lard**
Alloprevotella	-0.127 (0.537)	0.151 (0.483)	0.135 (0.550)
Anaerobiospirillum	-0.026 (0.915)	0.313 (0.178)	0.077 (0.748)
Bacteroides	0.109 (0.620)	-0.142 (0.497)	-0.018 (0.937)
Bifidobacterium	0.086 (0.658)	0.098 (0.614)	0.063 (0.758)
Blautia	-0.009 (0.964)	-0.063 (0.747)	0.058 (0.777)
Catenibacterium	0.009 (0.961)	0.024 (0.902)	0.189 (0.356)
Catenisphaera	-0.006 (0.974)	-0.079 (0.722)	0.111 (0.614)
Collinsella	0.037 (0.849)	-0.089 (0.648)	0.084 (0.682)
Faecalibacterium	-0.102 (0.626)	-0.131 (0.505)	-0.126 (0.576)
Helicobacter	0.385 (0.127)	0.123 (0.605)	0.034 (0.893)
Holdemanella	-0.007 (0.970)	-0.157 (0.416)	-0.091 (0.658)
Lachnoclostridium	-0.020 (0.918)	0.052 (0.789)	0.078 (0.706)
***Lachnospiraceae* NK4A136 group**	-0.042 (0.859)	-0.004 (0.986)	-0.015 (0.945)
Libanicoccus	-0.011 (0.957)	0.235 (0.247)	-0.088 (0.681)
Megasphaera	0.032 (0.880)	0.233 (0.252)	0.139 (0.517)
Negativibacillus	0.015 (0.939)	0.046 (0.813)	0.107 (0.610)
Parabacteroides	-0.061 (0.797)	-0.069 (0.791)	0.039 (0.874)
Peptoclostridium	0.018 (0.927)	-0.150 (0.436)	0.152 (0.458)
Peptococcus	-0.050 (0.795)	-0.029 (0.880)	0.045 (0.826)
***Prevotella* 9**	0.043 (0.823)	0.140 (0.477)	0.164 (0.435)
***Ruminiclostridium* 9**	0.135 (0.560)	-0.029 (0.894)	-0.047 (0.850)
***Ruminococcaceae* UCG-004**	0.135 (0.560)	-0.021 (0.931)	0.309 (0.212)
Sellimonas	0.092 (0.635)	0.070 (0.719)	-0.058 (0.780)
Slackia	0.026 (0.901)	-0.110 (0.569)	0.057 (0.792)
Solobacterium	-0.120 (0.535)	0.005 (0.981)	-0.063 (0.758)
Subdoligranulum	-0.202 (0.294)	-0.148 (0.445)	0.059 (0.780)
**unknown (Family *Atopobiaceae*)**	-0.155 (0.514)	-0.140 (0.555)	-0.049 (0.846)
**unknown (Family *Bifidobacteriaceae*)**	0.080 (0.704)	0.181 (0.409)	0.054 (0.822)
**unknown (Family *Eggerthellaceae*)**	-0.012 (0.954)	-0.116 (0.557)	-0.107 (0.604)
**unknown (Family *Erysipelotrichaceae*)**	0.093 (0.688)	-0.181 (0.409)	0.137 (0.543)
**unknown (Family Family XIII)**	-0.109 (0.620)	-0.296 (0.180)	-0.097 (0.693)
**unknown (Family *Lachnospiraceae*)**	-0.004 (0.982)	-0.076 (0.696)	0.065 (0.753)
**unknown (Family *Ruminococcaceae*)**	0.043 (0.866)	-0.201 (0.455)	-0.100 (0.673)
